# Access and Use of Digital Devices are Associated with Less Loneliness Over Time Among Older Adults

**DOI:** 10.1093/geroni/igaf122.3876

**Published:** 2025-12-31

**Authors:** Deborah Finkel, Anna Dahl Aslan

**Affiliations:** University of Southern California, Los Angeles, California, United States; University of Skövde, Skövde, Vastra Gotaland, Sweden

## Abstract

**Background:**

Loneliness, common among older adults, is a public health problem. The roots of loneliness are multifactorial, ranging from individual to structural factors. The digitalization of the society has the potential to influence older adults’ level of loneliness through individual factors, e.g., older adults’ access and use of digital devices, as the structure for how we interact and communicate has changed.

**Aim:**

to study the association between access and use of digital devices and loneliness over time.

**Methods:**

At baseline in 2004, (n = 785, mean age=70.1, SD = 11.2; 57.9% female; 100% European ancestry) and consecutively 4 more times over 10 years, participants in the longitudinal population-based Swedish Adoption/Twin Study of Aging responded to question about their access and use of computer and cellphone, and whether they are bothered by loneliness.

**Results:**

At baseline, access to cellphone and computer was 71% and 42% and daily use was 17% and 19%. By 2014, access increased to 86% and 64% and daily use increased to 46% and 37%. Repeated measures ANOVA indicated higher digital access (p = 0.001) and digital use (p = 0.054) as time-varying covariates were associated with less loneliness, while controlling for age, sex, and education.

**Conclusion:**

Among early adopters of digital devices both access and use are associated with less loneliness. The findings agree with a growing body of literature indicating that higher access and use of digital devices in old age potentially buffer against loneliness. Future studies need to explore the causal relation and control for a broader range of risk factors of loneliness.

